# Optimal Electrode Selection for Electrical Resistance Tomography in Carbon Fiber Reinforced Polymer Composites

**DOI:** 10.3390/ma10020125

**Published:** 2017-02-04

**Authors:** Luis Waldo Escalona Galvis, Paulina Diaz-Montiel, Satchi Venkataraman

**Affiliations:** 1 Computational Science Research Center, San Diego State University, 5500 Campanile Dr, San Diego, CA 92182, USA; lescalon@rohan.sdsu.edu; 2 Department of Aerospace Engineering, San Diego State University, 5500 Campanile Dr, San Diego, CA 92182, USA; pdiazmon@rohan.sdsu.edu

**Keywords:** effective independence, delamination, CFRP composites, sensor optimization, electrical resistance tomography

## Abstract

Electrical Resistance Tomography (ERT) offers a non-destructive evaluation (NDE) technique that takes advantage of the inherent electrical properties in carbon fiber reinforced polymer (CFRP) composites for internal damage characterization. This paper investigates a method of optimum selection of sensing configurations for delamination detection in thick cross-ply laminates using ERT. Reduction in the number of sensing locations and measurements is necessary to minimize hardware and computational effort. The present work explores the use of an effective independence (EI) measure originally proposed for sensor location optimization in experimental vibration modal analysis. The EI measure is used for selecting the minimum set of resistance measurements among all possible combinations resulting from selecting sensing electrode pairs. Singular Value Decomposition (SVD) is applied to obtain a spectral representation of the resistance measurements in the laminate for subsequent EI based reduction to take place. The electrical potential field in a CFRP laminate is calculated using finite element analysis (FEA) applied on models for two different laminate layouts considering a set of specified delamination sizes and locations with two different sensing arrangements. The effectiveness of the EI measure in eliminating redundant electrode pairs is demonstrated by performing inverse identification of damage using the full set and the reduced set of resistance measurements. This investigation shows that the EI measure is effective for optimally selecting the electrode pairs needed for resistance measurements in ERT based damage detection.

## 1. Introduction

Laminated carbon fiber reinforced polymer (CFRP) composite materials due to high stiffness to weight ratio are preferred in the aerospace industry. Health monitoring and prognosis of remaining useful life of CFRP composite structures require non-destructive evaluation (NDE) techniques to inspect the material. Current NDE methods that have been used to inspect composites include ultrasonic scanning, X-ray imaging, acoustic emission and thermography [1].

In CFRP composites, the carbon fibers are electrically conductive and the matrix material is an insulator. The contact between fibers due to fiber waviness provides electrical conduction in the direction normal to the continuous fibers. Failure modes in CFRP include intralaminar matrix cracking and interlaminar delamination cracks. The presence of cracks due to delamination or matrix cracking break these contacts through which the electric current flows and increases the electrical resistance. This allows using the intrinsic electrical resistance change to sense damage in a composite. Electrical Resistance Tomography (ERT) is an NDE approach which has become increasingly popular in the context of structural health monitoring. In ERT, the changes in electrical resistance are measured from the composite laminate boundaries and the damage is characterized through inverse identification techniques. A number of publications have appeared in the last few decades that demonstrate the use and applicability of ERT methods for structural health monitoring in laminated fiber reinforced polymer (FRP) composites [2,3].

Inverse identification of delamination crack location and size in ERT requires numerical optimization that minimizes the difference between the measured and the model predicted resistances, with the damage location and size as its variables. This optimization becomes prohibitively expensive computationally for composite materials since it requires numerical finite element analysis models. Damage identification based on electrical resistance change using surrogate modeling based on response surface methods [4,5,6] or neural networks [7] has been presented to reduce model complexity.

An essential feature in the design of a sensing system for damage detection is to ensure that the obtained measurements are able to identify damage with good sensitivity and distinguishability for every considered case. However, this requires a large number of sensors which is not possible in practical applications since the number of measurements are limited by the hardware restrictions such as sensor availability, data channels and sampling time. This necessitates methods for optimum selection of electrodes that provide high detectability and distinguishability. In ERT for damage characterization in composites, this optimization translates into minimizing the number of electrode sensing combinations used for resistance measurements, preserving accuracy in the characterization of delamination location and size.

Optimum sensor selection has been previously explored in the structural vibration field. The Effective Independence (EI) measure was introduced by Kammer [8] for optimum placement of acelerometers in space structures to identify a given set of vibration modes. This work presented a procedure for the reduction of sensor locations used in modal analysis in large structures based on ranking the sensor locations according to their contribution to the linear independence of the target modal partitions [8].

This paper extends the approach used by Kammer by using the EI measure to rank and classify the measuring electrode combinations in ERT. The use of effective independence ranks electrode pairs for resistance measurements according to their contribution to the independence of the damage states, thus determining the minimum number of required electrodes to characterize such states. Preliminary considerations and assumptions for using EI for the ERT problem on composites are presented. Experiments simulated by numerical finite element analyses are used to demonstrate the application of using EI in selecting the electrode configurations for ERT based detection of delamination in composite laminates. The verification of the accuracy is demonstrated using inverse identification optimizations.

## 2. Electrical Resistance Tomography in CFRP Composite Laminates

Carbon Fiber Reinforced Polymer (CFRP) laminates exhibit highly orthotropic electrical conductivity properties along their fiber, transverse and thickness directions. Electrical conductivity in CFRP laminates along the fiber direction is the highest. Since the polymer matrix usually behaves as an insulator material, the electrical conductivity in the transverse and thickness directions should be zero or negligibly small for perfect unidirectional CFRP laminates. However, the fiber waviness in real composites lead to fiber-to-fiber contact creating a network that allows the electrical current to flow in the transverse to fiber direction.

The presence of a resin-rich region between plies in laminated composites made from pre-preg materials further lowers the electrical conductivity in the thickness direction compared to the inplane transverse to fiber direction [5]. As a result, the ratio of electrical conductance in the the fiber direction ($\sigma_{0}$) to the transverse to fiber direction ($\sigma_{90}$) is approximately $\sigma_{0}/\sigma_{90}=10^{3}$, and the electric conductivity ratio of the fiber direction to the thickness direction ($\sigma_{t}$) is approximately $\sigma_{0}/\sigma_{t}=10^{4}$ [9]. Intralaminar transverse cracks that occur inside a ply layer and intralaminar delamination cracks that occur between the plies break the fiber-contact network between plies, which further decreases the electrical conductivity (increases the electrical resistance ) in the inplane transverse and out of plane directions.

**Figure 1:**
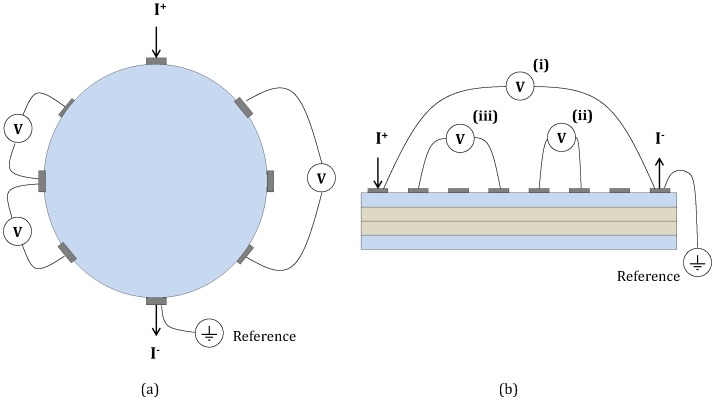
Electrical Resistance Tomography (ERT): (**a**) General example; (**b**) in a laminate.

ERT based damage detection applies electric current resulting from an external electric potential at electrode pairs on a specimen surface and measures the electric potential difference between electrode pairs [10] (Figure 1a). Analogously, for ERT on a laminate, electric potentials can be measured [11] (Figure 1b) between: (i) the electrode pair on which electric current is injected, (ii) consecutive electrodes or (iii) a couple of electrodes following a given separation or skip [10]. Electrical resistance values between the electrode pair through which excitation is applied can be determined using Ohm’s law. These values can be obtained before and after delamination occurs in the composite, and the change in resistance is used to measure the damage.

In order to measure electrical resistance on a composite laminate, many authors have placed electrodes on both top and bottom surfaces using different configurations [4,9,12]. Delamination and matrix damage detection using ERT is challenging because these damage modes are perpendicular to directions in which the conduction is already low. This requires investigation of methods to optimize sensors to increase sensitivity of the measurements that leads to more accurate damage detection and identification.

## 3. Effective Independence for Optimum Sensor Location Selection

Kammer [8] presented a methodology for selecting an optimum set of sensor locations for the identification of a set of target vibration modes using finite element models. Based on modal representation from structural dynamics, the *m*-dimensional vector $u_{s}$ with the sensed output displacements describing the physical degrees of freedom (DOF) of a structure is related to the *n*-dimensional vector *q* of target modal coordinates (modal DOFs) through the eigenmodes of the system given by the $m_{x}n$ matrix $\Phi_{s}$:

(1) $$u_{s}=\Phi_{s}q.$$

In Equation (1), $\Phi_{s}$ represents the sensing locations in its rows and the target modes in its columns. For $u_{s}$ with added stationary Gaussian white noise N with variance $\sigma_{s}^{2}$:

(2) $$u_{s}=H\left(q\right)+N=\Phi_{s}q+N,$$

the covariance matrix P of the estimate error for an efficient unbiased estimator is given by Equation (3). For H(q) equal to $\Phi_{s}q$, *P* can be expressed in terms of $\Phi_{s}$ (Equation (4)) which equals the inverse of the Fisher information matrix *Q*. Therefore, minimizing *P* implies the maximization of *Q*:

(3) $$P=E\left[\left(q-\hat{q}\right){\left(q-\hat{q}\right)}^{T}\right]={\left[{\left(\frac{\partial H}{\partial q}\right)}^{T}{\left[\sigma_{s}^{2}\right]}^{-1}\left(\frac{\partial H}{\partial q}\right)\right]}^{-1},$$

(4) $$P={\left[\Phi_{s}^{T}{\left(\sigma_{s}^{2}\right)}^{-1}\Phi_{s}\right]}^{-1}=Q^{-1}.$$

By assuming that the measurement noise is uncorrelated and possesses identical statistical properties for each sensor, *Q* can be expressed as shown in Equation (5). Matrix $A_{0}$ is an equivalent Fisher information matrix [13] expressed in terms of Equation (6),

(5) $$Q=\frac{1}{\sigma_{s}^{2}}\Phi_{s}^{T}\Phi_{s}=\frac{1}{\sigma_{s}^{2}}A_{0},$$

(6) $$A_{0}\Rightarrow \sum_{i=1}^{m}Q_{i}=\sum_{i=1}^{m}{\Phi_{si}}^{T}\Phi_{si}.$$

In Equation (6), *m* is the number of candidate sensors and $\Phi_{si}$ is the *i*th row of the target mode partition matrix related to the *i*th candidate sensor location. Therefore, as sensors are removed from the candidate set, information is deleted from Q [13].

For a matrix $\Phi_{s}$ of eigenmodes with column vectors linearly independent, matrix $A_{0}$ will have real positive eigenvalues and orthonormal eigenvectors in matrix *Ψ* (Equation (7)). The eigenvectors $\psi_{j}$ form the absolute identification space [8],

(7) $$[A_{0}-\lambda I]\Psi =0.$$

**Figure 2:**
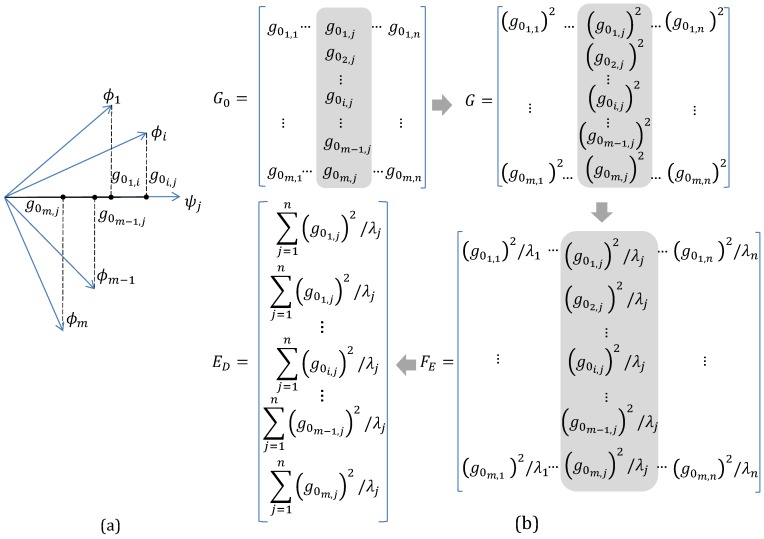
Determination of the Effective Independence (EI) distribution for the sensor set used for the target modes: (**a**) Projection of row vectors of $\Phi_{s}$ on a given column vector $\psi_{j}$ of *Ψ*; (**b**) Operations involving matrices $G_{0}$, *G* and $G_{\lambda }$ for the generation of vector $E_{D}$.

Postmultiplying matrix $\Phi_{s}$ by matrix *Ψ* results in a matrix $G_{0}$ (Equation (8)) in which the *j*th column vector contains the orthogonal projection of each row vector of $\Phi_{s}$ on the *j*th eigenvector (column vector) in *Ψ* such that the *i*th component of this vector contains the projection of the *i*th row vector from $\Phi_{s}$ (Figure 2a),

(8) $$G_{0}=\Phi_{s}\Psi .$$

Squaring each entry of matrix $G_{0}$ (Equation (9)) results in matrix *G* (Figure 2b). For this matrix, the *i*th component of its *j*th vector contains the contribution of the *i*th sensor location to the associated *j*th eigenvalue in *Ψ* [8].

(9) $$g_{i,j}={\left(g_{0_{i,j}}\right)}^{2}\Rightarrow G$$

Postmultiplying matrix *G* by the inverse of matrix *λ* with the eigenvalues of $A_{0}$ (Equation (10)) divides every column of *G*, related to the *j*th eigenvector in *Ψ*, by its eigenvalue which gives equal importance to each direction within the identification space [8]. The result of this product is matrix $F_{E}$ (Figure 2b) with the fractional contribution of the *i*th sensor location of the *j*th eigenvalue [8],

(10) $$F_{E}=G\lambda^{-1}.$$

Adding the entries within each row of matrix $F_{E}$ yields column vector $E_{D}$ (Figure 2b) with the effective independence (EI) distribution of the sensor set [8]. Vector $E_{D}$ is alternatively formulated as the diagonal of the matrix *E* shown in Equation (11) [8],

(11) $$E=\Phi_{s}{\left[{\Phi_{s}}^{T}\Phi_{s}\right]}^{-1}{\Phi_{s}}^{T}.$$

## 4. Problem Formulation and Solution

### 4.1. Effective Independence Applied to Electric Resistance Tomography Measurements

Applying the EI approach to ERT requires modification of the modal analysis formulation to electrical resistance measurements. In ERT, the equivalent for the sensed output displacements $u_{s}$ (Equation (1)) are the electrical resistance changes measured at electrode locations on the boundaries in the presence of specific delamination. The candidate sensing locations correspond to all the possible combinations of excitation electrode pairs from which an electric resistance can be measured. The vector of electrical resistance changes due to a damage case (delamination) is formulated analogously to modal representation in structural dynamics (Equation (12)), namely,

(12) $$\Delta r=\Phi_{s}\chi ,$$

where the vector Δr represents the total set of *m* resistance change measurements on a given laminate under damage with respect to an undamaged condition, and matrix $\Phi_{s}$ represents a set of (column) vectors describing a new basis spanning the resistance changes (contained in Δr) as a function of damage associated to the vector *χ* containing the contribution of each vector of $\Phi_{s}$ to the measured resistance Δr.

Equation (12) can be generalized for multiple damage cases by determining a $m_{x}n$ matrix $\Phi_{s}$ with the vector basis for the electrical resistance change space. The basis $\Phi_{s}$ can be obtained using proper orthogonal decomposition of the governing equations for ERT. Instead, a numerical surrogate method is proposed here that uses the forward solutions to the governing equation for a finite sample of *n*-realizations from a space of all possible damages, and the resistance changes at all possible sensor locations are determined. This information is assembled into a ΔR matrix, with each column representing the resistance change due to a particular damage state. The ΔR matrix can then be expressed using a finite basis as:

(13) $$\Delta R=\Phi_{s}X.$$

The basis in $\Phi_{s}$ can be obtained by computing the eigenvalues of the covariance matrix $\Delta R\Delta R^{T}$ using Principal Component Analysis (PCA) or Singular Value Decomposition (SVD). Since ΔR is often ill conditioned, SVD can be used to reduce ΔR and thus an equivalent matrix $\Phi_{s}$ can be obtained. In SVD, ΔR is factorized as:

(14) $$\Delta R=U\Sigma V^{T}.$$

By doing a reduction on the factorization terms based on retaining the largest singular values (in *Σ*), a reduced $\Delta R_{r}$ matrix results:

(15) $$\Delta R_{r}=U_{r}\Sigma_{r}{V_{r}}^{T},,$$

where $U_{r}\Sigma_{r}=\Phi_{s}$ and $X={V_{r}}^{T}$.

Once a $\Phi_{s}$ is computed using the factorization from Equation (15), a matrix *E* is obtained using Equation (11), and, from its main diagonal, the EI coefficients are assigned to vector $E_{D}$. Accordingly, the *i*th component of vector $E_{D}$ ranks the contribution from the electrode combination related to the *i*th row of matrix ΔR to the effective independence in the identification of damage cases. Hence, electrode combinations can be ranked using their associated $E_{Di}$, allowing for a reduction in the number of sensing combinations.

Reducing the number of electrode combinations using the EI measures can be attained in a systematic and iterative fashion [13]. For the reduction, the effective independence (EI) values of $\Phi_{s}$ are computed and then the row associated with the lowest EI value is eliminated from $\Phi_{s}$. These actions are repeated until the determinant of the updated matrix $A_{0}$ approaches zero ($\epsilon =10^{-9}$). Thereafter, the number of remaining electrode combinations never exceeds the amount of retained singular values in $\Sigma_{r}$.

For the quantitative assessment of the EI measure based optimal selection of electrode combinations, this work uses a comparison of inverse identification of delamination with the full set of electrodes to the optimally reduced set of electrodes. Surrogate based optimization is used for the inverse identification of damage cases. The surrogate model is constructed from the definition of a design space and the solution of a forward problem consisting of 2D finite element models. The next section presents modeling considerations for the ERT forward problem and the EI measure results along with the surrogate based inverse identification are presented later.

### 4.2. Solving the Forward Problem in ERT to Simulate Resistance Measurements

Damage detection in a composite laminate using ERT requires solving the forward problem describing the electrical response in a domain with conductivity field inside a material as a function of a given damaged parameters. The governing differential equation for the voltage distribution *u* in a body with a conductivity field ***σ*** under the spatial coordinates $\bar{r}$ is given by the following Laplace’s equation and associated boundary conditions (BC) [14,15,16]:

(16) $$\nabla \;\cdot \;\sigma \nabla u=0,\;\bar{r}\in \Omega ,$$

(17) $$\int_{el}\sigma \frac{\partial u}{\partial \bar{n}}dS=I,\;\bar{r}\in el,\;l=1,2,\ldots ,L,$$

(18) $$\sigma \frac{\partial u}{\partial \bar{n}}=0,\;\bar{r}\in \partial \Omega \backslash \;U_{l=1}^{L}\;e_{l},$$

(19) $$u+\xi \sigma \frac{\partial u}{\partial \bar{n}}=U_{l},\;\bar{r}\in e_{l},\;l=1,2,\ldots ,L.$$

Equation (17) establishes that the integral of the current density over the *l*th electrode (surface) is equal to the electric current $I_{l}$ through the electrode ($\bar{n}$ is the outward unit normal). Equation (18) implies no current flows through the electrode-free boundaries. Finally, Equation (19) represents the behavior at the interface of the electrode, and the medium where an infinitely thin layer with a contact surface resistance *ξ* is considered [14]. When electric contact between an electrode and the target surface is perfect, the voltage drop due to contact resistance *ξ* term is neglected and the electrical potential *u* under electrode $e_{l}$ is equal to $U_{l}$.

In addition, for a proper problem formulation, the currents $I_{l}$ need to satisfy the charge conservation condition and a potential reference must be set to zero for uniqueness in the solution [14]

For orthotropic fiber reinforced composites, the material properties vary in each layer due to ply orientation and the introduction of cracks/delaminations within the laminate introduces free surfaces within the domain. The solution of the governing equation formulated above requires numerical methods such as the Finite Element Method (FEM). The effective resistance R(σ) across any electrode pair, through which a current I flows, can be computed from the measured voltage V using Ohm’s Law as:

(20) V=R(σ)I,

where V and I contain voltage measurements (between electrodes) and electric currents, respectively. An approximation of the conductivity distribution is given by ***σ***. It is possible to use the governing Equation (16) along with Equation (20) to describe the physics of a composite laminate under electric load.

**Figure 3:**
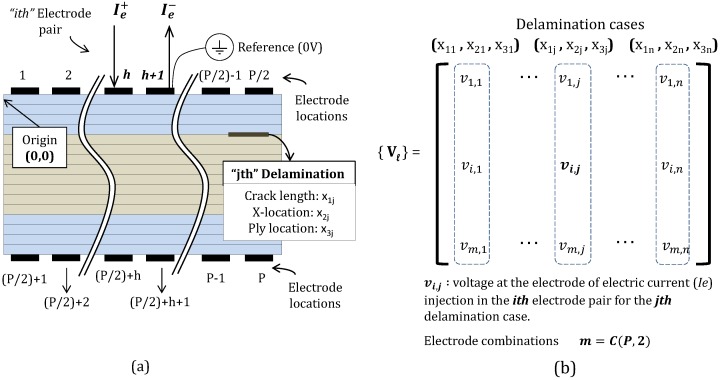
Formulations for the resistance measurements. (**a**) Schematic representation of the 2D model for the composite laminate (case of $[0_{4}/90_{4}{]}_{s}$); (**b**) Electric potential matrix for a composite under damage conditions.

The present work uses simulated measurements obtained from solving a numerical model of the problem. A simplified 2D in the plane model for the laminate that spans the thickness and length of the laminate is considered (Figure 3). The specimen, electrodes and the embedded delamination are all assumed to span the entire length in the out-of-plane direction. The Finite Element Analysis (FEA) model is used to solve the equations governing the electrical current density flow in a composite laminate with orthotropic materials.

The 2D finite element model is used to analyze the composite laminate with embedded delamination for a given current path due to an electrode combination (where current $I_{e}$ is injected) and obtain the resulting voltage distribution. Figure 3a shows a schematic exemplifying the composite laminate with *P* electrodes, where the electrical current is injected between electrodes *h* and h+1 forming the *i*th pair. For a laminate with specified properties and *P* electrodes evenly distributed on both the top and bottom surfaces (Figure 3a), electric current is applied on all possible electrode pair combinations. The number of combinations *m* of choosing two electrodes from *P* electrodes equals the combinatorial coefficient ${}_{P}C_{2}$. The finite element analyses are repeated for *n* different damage cases. For each case, the obtained voltage is the electric potential difference between the electrode pair used for electric current injection. Therefore, *m* electric potential values are computed for each damage case forming a column vector of matrix $V_{l}$.

The matrix $V_{l}$ (Figure 3a) has *m* rows corresponding to the electrode pair combinations and *n* columns for the damage cases (Figure 3b). Each damage case is represented by: delamination length $\left(x_{1}\right)$, horizontal location $\left(x_{2}\right)$, and vertical location $\left(x_{3}\right)$ (Figure 3a). Every matrix entry contains the voltage on the electric current injection electrode. Analogously, a control matrix $V_{0}$ representing the voltage values on the laminate when no damage is present is determined. This matrix is the same size of $V_{l}$ but with repeated column vectors representing no damage state. The matrix of resistance changes ΔR calculated as $\left(V_{l}-V_{0}\right)/I_{e}$ represents the change in electric resistance measured between the electrode pair combinations given the delamination cases. This matrix summarizes the electric resistance change in the laminate under the different considered cases.

The 2D finite element models consisting of 16 ply laminates were created and solved using a commercial software (ANSYS^®^ Academic Research, Release 15.0.7, Canonsburg, PA, USA). Delamination cracks were created using doubly-defined nodes at the crack interface to represent for free surfaces. The domain was meshed with eight node quadrilateral planar electric elements (PLANE 230). The element size was selected based on a mesh convergence study which used electric potential difference between excitation electrode pairs located at a center location, for a delamination crack located at two plies under the injection electrode. Different mesh sizes tested until convergence were observed. The converged element mesh size was found to be 0.125 mm, which completely spans through the thickness of each ply. This resulted in a total number of 27,044 elements for the model.

**Figure 4:**
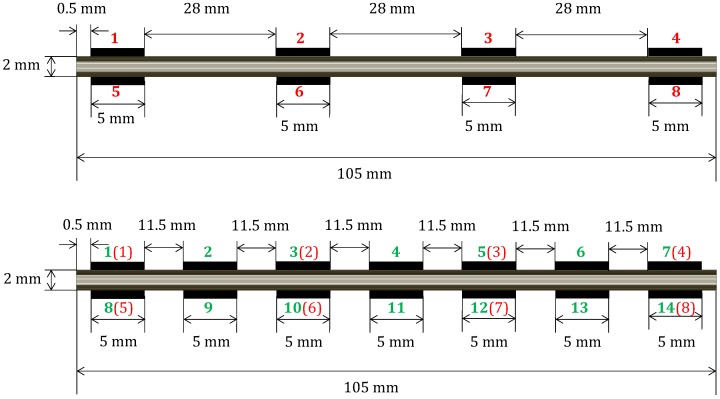
Laminate and electrode model used for the finite element analyses. Case of $[0_{4}/90_{4}{]}_{s}$ laminate with eight electrodes (**top**) and 14 electrodes (**bottom**). Numbering for eight electrodes (red) and 16 electrodes (green) with its 8-electrode equivalence in parentheses.

Surface electrodes were modeled as thin sheets of silver and sets of 8 and 14 were evenly distributed on the top and bottom surfaces of each laminate model (Figure 4). For resistance measurements, this results in a total of 28 (C28) electrode pair combinations for eight electrodes and 91 (C214) combinations for 14 electrodes. A low electric current of 30 mA is applied on the injection electrode and the reference electrode which is set to 0 V.

For the FEA model, orthotropic electrical properties were used for the $[0_{4}/90_{4}{]}_{s}$ and $[{\left(0/90\right)}_{4}{]}_{s}$ cross-ply laminate models, whereas electrode material is considered isotropic. The resistivity values used correspond to a graphite epoxy composite lamina of 62% fiber volume fraction [5]. Table 1 lists the conductivity values used for the plies in the composites for the FEA modeling. A conductivity of 62.9 S/m is assigned to the electrode material.

### 4.3. Selection of Optimum Set of Electrode Pairs for ERT Based NDE Using Effective Independence

Using EI to determine the optimum set of electrode pairs for damage identification using ERT first requires the determination of the resistance change matrix after analyzing a set of damage cases set by the three previously defined design variables related to the crack: $x_{1}$, $x_{2}$ and $x_{3}$. Variable $x_{1}$ assumed one of six possible length values (in mm) from the set: [5, 10, 15, 20, 25, 30]. For $x_{2}$, horizontal crack center locations (in mm) for damage assumed one of the nine possible values: [19.5, 27.75, 36, 44.25, 52.5, 60.75, 69, 77.25, 85.5]. Variable $x_{3}$, crack vertical location, assumed values from 1 to 15 associated with the intralaminar locations. A full factorial design of the space defined by the variables ${x_{1}}_{x}{x_{2}}_{x}x_{3}$ resulted in 810 cases (6 × 9 × 15).

The 8-electrode case (Figure 4, top) is a subset of the 14-electrodes case (Figure 4, bottom). Therefore, only simulations for the 14-electrodes case were performed. The corresponding equivalence in the electrodes numbering when comparing data for eight electrodes to 14 electrodes is shown in parentheses (red) in Figure 4. Thus, for 810 cases, a total of 73,710 (91 × 810) FE analyses for every given stacking sequence were necessary.

For each stacking sequence, resistance change matrices $\Delta R_{28\times 810}$ and $\Delta R_{91\times 810}$ were computed for eight and 14 electrodes. The SVD based reduction of the ΔR matrix was performed by iteratively eliminating singular values from *Σ* in the factorization shown in Equation (14) until the scalar representing the relative change ΔM between the reconstructed and the original ΔR matrices exceeded 2% as defined in Equation (21):

(21) $$\Delta M=100\times \frac{∥U\left(\Sigma -\Sigma_{r}\right)V^{T}∥}{∥\Delta R∥},$$

where $\Sigma_{r}$ is the diagonal matrix with the *r* retained singular values after each iteration. The equivalent $\Phi_{s}$ shown in Equation (15) is formed by the matrix $U_{r}$ containing the *r* left singular vectors after the reduction. Using $U_{r}$ as $\Phi_{s}$ provides a matrix with a set of column vectors from the spanning set of ΔR, which results in a matrix $A_{0}$ with condition number equal to 1.

### 4.4. Validation of Electrode Pair Reduction Using Inverse Identification

The validity of using EI measures to reduce the amount of resistance measurements for damage detection is demonstrated by comparison of inverse identification results obtained from the full set and the reduced set of measurements from the models. Inverse identification minimizes the $L_{2}$ norm of the difference between the model predicted resistances and the measured resistance with damage descriptors on variables. The optimization problem for this unconstrained nonlinear multivariate minimization problem with bound constraints on the variables is presented in Equation (22):

(22) $$\underset{x_{i}^{L}<x_{i}<x_{i}^{U},\;i=1,2,3}{\underset{x_{1},x_{2},x_{3}\;}{Min\;}}f\left(x\right)=\sum_{i=1}^{ndim}{\left(y-\hat{y}\right)}^{2},$$

where the design variables $x_{i}$ describe the delamination size and location, ndim is the number of resistance values of each design point, and *y* and $\hat{y}$ are the vectors of measured and model calculated resistance values, respectively. The minimization was performed using commercial software (MATLAB and Optimization Toolbox Release 2016a, The MathWorks, Inc., Natick, MA, USA). Since the optimization problem formulated above is a mixed variable problem (continuous in $x_{1},\;x_{2}$ and discrete in $x_{3}$), a genetic algorithm (GA) was used. The GA optimization used an initial parent population of 80 individuals, with child populations obtained using cross-over and mutation operators based on an elitist strategy. Parent population of 60 and 80 individuals along with mutation rates of 1% and 5% were also evaluated. The GA was run for a maximum of 300 generations and terminated if there were no improvements after 200 generations. The algorithm was run for each population size, in order to evaluate the optimum designs. The lower and upper bounds for the design variables were defined as in Table 2. The optimization process was repeated three times for each case and the optimization, providing the lowest number of cases with high relative errors was taken.

A krigging [17,18] approximation was fitted to resistance change values chosen after the full factorial (${x_{1}}_{x}{x_{2}}_{x}x_{3}$) Design of Experiments (DOE) with 810 points [19].

## 5. Results and Discussion

### 5.1. Effective Independence Applied to ERT

Table 3 groups EI values for the delamination cases in the ${\left[0_{4}/90_{4}\right]}_{s}$ and $[(0/90{)}_{4}{]}_{s}$ laminates with eight electrodes. Both laminates show similar behavior. The highest EI contributions are found for vertically opposed electrodes close to the center of the specimen. High EI contributions are also on those opposing ones near the edges and consecutive electrode pairs on the same surface by the center. Consecutive electrodes near the edges and those diagonally the closest have medium contributions. The lowest EI contributions are found on electrodes with nonzero skips and large diagonal separation.

**Figure 5:**
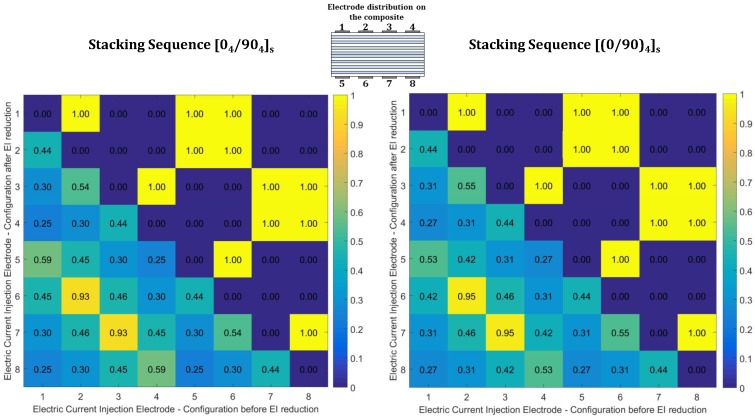
Graphical representation of effective independence values before and after electrode combination reductions for $[0_{4}/90_{4}{]}_{s}$ (**left**) and $[(0/90{)}_{4}{]}_{s}$ (**right**) composite laminates with eight electrodes (the above diagonal indicates retained electrodes).

**Figure 6:**
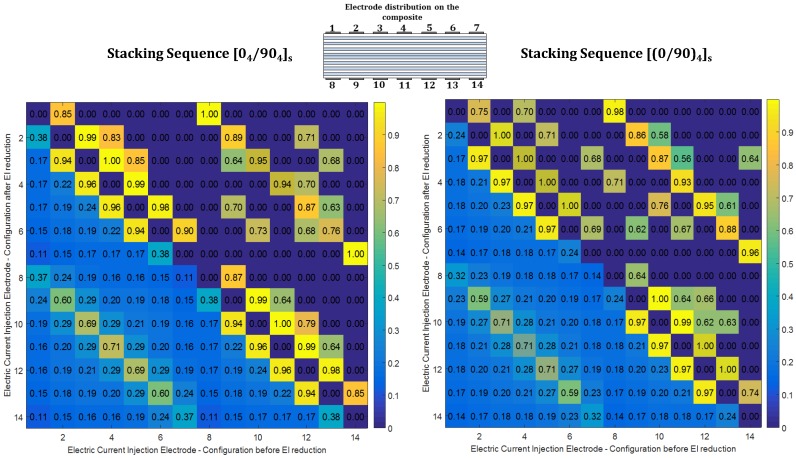
Graphical representation of effective independence values before and after electrode combination reductions for $[0_{4}/90_{4}{]}_{s}$ (**left**) and $[(0/90{)}_{4}{]}_{s}$ (**right**) composite laminates with 14 electrodes. (the above diagonal indicates retained electrodes).

Figure 5 and Figure 6 present heatmaps for graphical comparison of the EI values before and after reduction for both stacking sequences. The heatmaps were plotted using a toolbox developed for MATLAB [20]. The diagrams present EI values before reduction in their area below the diagonal and the EI values after reduction in the area above the diagonal. For an electrode pair, a first electrode value set on the abscissa and the second one on the ordinate gives an EI value before reduction. On the other hand, a first electrode value set on the ordinate and the second on the abscissa gives an EI value after reduction.

Figure 6 shows that, for 14-electrodes cases, both stacking sequences find the highest contributions in combinations of consecutive electrodes on the same side followed by combinations of opposing electrodes.

Electrode reduction using EI measures resulted in an optimum selection of 12 from 28 possible pairs for eight electrodes for both stacking sequences. From the original set, those combinations with EI contributions larger than 0.9 are preserved, whereas the other retained combinations do not follow the order in Table 3. Figure 5 shows that all EI based ranks of electrode combinations are qualitatively the same for both stacking sequences.

In the 14-electrode cases, the elimination process resulted in an optimum selection of 32 and 34 electrode pairs from the complete set of 91 pairs for the ${\left[0_{4}/90_{4}\right]}_{s}$ and $[(0/90{)}_{4}{]}_{s}$ laminates, respectively. Figure 6 shows the heatmaps of EI values for the 14-electrode cases. For the EI based reduction on the $[0_{4}/90_{4}{]}_{s}$ laminate, consecutive electrodes hold the highest ranks followed by electrode pairs opposing each other. For the $[(0/90{)}_{4}{]}_{s}$ laminate, opposite sensing pairs generally show the highest ranks followed by consecutive electrode combinations after reduction takes place.

### 5.2. Inverse Identification of Damage in the Composites

The errors are computed by taking the difference between the inverse identification prediction and the actual values. To obtain relative errors, these values were normalized as described below.

**Figure 7:**
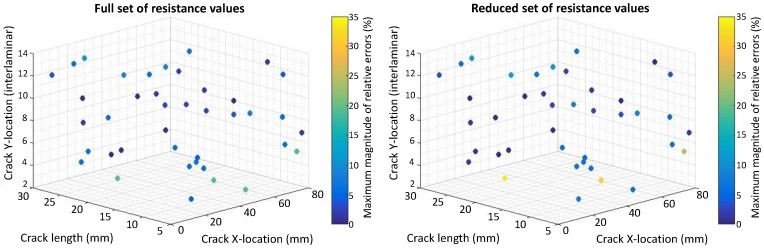
Scatter plot with maximum magnitude of relative errors obtained at the 40 test points used for inverse identification in a $[0_{4}/90_{4}{]}_{s}$ laminate using eight electrodes and considering: (**left**) Full resistance measurement set; (**right**) Reduced resistance measurement set using EI.

**Figure 8:**
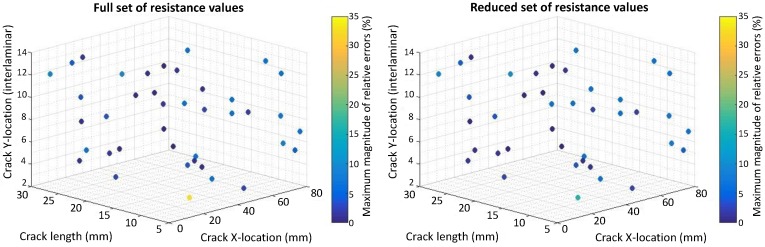
Scatter plot with maximum magnitude of relative errors obtained at the 40 test points used for inverse identification in a $[0_{4}/90_{4}{]}_{s}$ laminate using 14 electrodes and considering: (**left**) Full resistance measurement set; (**right**) Reduced resistance measurement set using EI.

**Figure 9:**
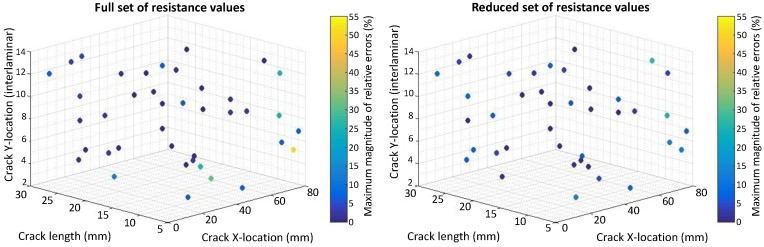
Scatter plot with maximum magnitude of relative errors obtained at the 40 test points used for inverse identification in a $[(0/90{)}_{4}{]}_{s}$ laminate using eight electrodes and considering: (**left**) Full resistance measurement set; (**right**) Reduced resistance measurement set using EI.

**Figure 10:**
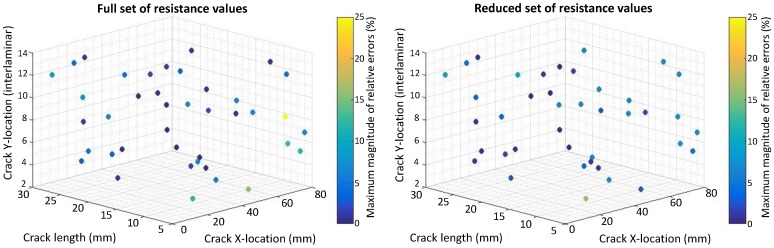
Scatter plot with maximum magnitude of relative errors obtained at the 40 test points used for inverse identification in a $[(0/90{)}_{4}{]}_{s}$ laminate using 14 electrodes and considering: (**left**) Full resistance measurement set; (**right**) Reduced resistance measurement set using EI.

The relative errors (REs) are computed for the crack size, horizontal and vertical locations by comparing them to the values found by the inverse identification optimization to the actual values for 40 test cases obtained by a Latin Hypercube Sampling (LHS). The REs for crack size are calculated relative to the electrode center to center spacing equal to 33 mm and 16.5 mm for the case of eight and 14 electrodes, respectively. The REs for the horizontal spacing are calculated relative to the range of $x_{2}$ used for the DOE points $\left({x_{2}}^{U}-{x_{2}}^{L}=69-19.5=49.5\right)$ mm. The REs for vertical locations are computed relative to the total number of plies (16). Figure 7, Figure 8, Figure 9 and Figure 10 present the maximum magnitude of the RE obtained after inverse identification of the 40 design points. They present plots of each laminate ( $[0_{4}/90_{4}{]}_{s}$ or $[(0/90{)}_{4}{]}_{s}$ ) for eight and 14 electrodes before and after reduction is done using EI.

The inverse identification optimization results for a $[0_{4}/90_{4}{]}_{s}$ laminate and eight electrodes (Figure 7) with the full and reduced optimum sets of resistance measurements showing large relative errors (>10%) at six out of 40 test cases in the full set and six cases when the reduced set of electrode pairs was considered, considering REs larger than or equal to 5% results in 13 cases for the full set and 14 cases in the reduced one. In the case of 14 electrodes in the $[0_{4}/90_{4}{]}_{s}$ laminate (Figure 8), eight cases present large errors for the full set of electrode pairs against seven cases for the reduced set. Both the full set and the reduced set of electrode pairs presented 16 cases with REs larger than or equal to 5%.

The inverse identification optimization results for the $[(0/90{)}_{4}{]}_{s}$ laminate and eight electrodes (Figure 9) with the full and reduced optimum sets of resistance measurements showed five cases with large RE (>10%) for the full set and seven cases out of the total 40 when the reduced set of pairs was considered. For the full set, 19 cases showed RE larger than or equal to 5%, whereas, for the reduced set, 21 cases in this condition were found. In the case of 14 electrodes in the $[(0/90{)}_{4}{]}_{s}$ laminate (Figure 10), four cases present large REs in the full set of electrode pairs and only two cases do for the reduced set. Nonetheless, 15 cases show RE larger than or equal to 5% when the full set of electrode combinations is used, and this number increases up to 19 when reduction takes place.

In order to quantify the effectiveness of using EI on the electrode combinations, the root mean square (RMS) between the reduced electrode pair set and the full set of relative errors at each test point is computed ($RE_{RMS}$) according to Equation (23):

(23) $$RE_{RMS}=\sqrt{\frac{\sum_{i=1}^{N_{T}}{\left(\%RE_{reduced}-\%RE_{full}\right)}^{2}}{N_{T}},}$$

where $N_{T}$ is the number of test points and $\%RE_{reduced}$ and $\%RE_{full}$ are the maximum relative errors at the *i*th test point for the reduced electrode pair set and full electrode pair set, respectively. The maximum relative error is chosen from one of the three $x_{j}\left(j=1,2,3\right)$ space variables according to Equation (24):

(24) $$RE\left(\%\right)=\underset{j\;}{Max\;}\left|\left(\frac{x_{j}-{\hat{x}}_{j}}{{x_{j}}^{ref}}\right)\right|\times 100\%,$$

The RMS for the $[0_{4}/90_{4}{]}_{s}$ laminate using eight electrodes was 8.75% and when using 14 electrodes was 4.76%. On the other hand, the $[(0/90{)}_{4}{]}_{s}$ results in RMS values of 4.20% for eight electrodes and 3.01% for 14 electrodes. In general, these results suggest that, as the number of electrodes increases, the relative errors in the measures before and after applying EI reduction on the electrode pair set approach each other. This latter aspect is more significant in the $[0_{4}/90_{4}{]}_{s}$ laminate, where, in addition to when using eight electrodes, the reduced set presents 25 test points where the absolute value of their maximum error is larger than for the full set, and this value significantly reduces to 15 when the number of electrodes increases to 14. This reduction is not observed in the $[(0/90{)}_{4}{]}_{s}$ laminate where actually the number of cases increases from 16 for eight electrodes to 18 when using 14 electrodes, which can be related to the light variation observed in the RMS values.

Table 4 summarizes the inverse identification results. Three metrics are applied to the maximum errors at the test points: Root Mean Square Error (RMSE) over the set of points, and maxima of the maximum errors and number of cases with absolute values of error larger than 10%. For the $[0_{4}/90_{4}{]}_{s}$ laminate, RMSE values suggest that identification deviations decrease when increasing the number of electrodes. The maxima of the errors also decreased accordingly but not the number of cases with errors larger than 10%. Applying effective independence reduction results in a decrease of the RMSE values and the maxima for eight electrodes, but the opposite when 14 electrodes are used. The number of cases with large errors (>10%) never changed after reduction for this layup.

For the $[(0/90{)}_{4}{]}_{s}$ laminate, deviations do not follow a trend when increasing the number of electrodes, as evidenced from the RMSE values. The RMSE values slightly increase when increasing the number of electrodes for the full electrode set, but the opposite occurs with reduced set cases. The same occurs with the maxima. The number of cases with large errors always decreases when moving from eight to 14 electrodes with and without reduction. With regard to the sensing pairs reduction for a given number of electrodes, applying effective independence results in an increase of the RMSE values, the maxima and the number of failed cases when eight electrodes are used, which means worsening of the fit. The opposite behavior is observed with 14 electrodes.

## 6. Conclusions

This paper has presented an approach for optimum selection of electrodes or measurements for inverse identification of delamination damage via Electrical Resistance Tomography. The optimum electrode selection is based on the Effective Independence measure originally proposed for vibration modal identification. A Singular Value Decomposition was needed to apply the EI measure to resistance measurements in Electrical Resistance Tomography damage detection. The SVD also offered additional benefits in dimensional reduction. The EI based electrode selection was performed. The procedure was assessed through comparisons of inverse identification optimization performed using a full set of measurements and a set of reduced measurements (as identified by the EI measure) on two different stacking sequences for two sets of excitation/sensing electrodes. The results indicate that the effective independence based procedure offers a viable way to perform optimum electrode selection for ERT based damage detection.

## Figures and Tables

**Table 1:** Orthotropic conductivity *σ* values used for the finite element analysis models [4].

Fiber Direction ($\sigma_{0}$ [S/m])	Transverse to Fiber Inplane ($\sigma_{90}$ [S/m])	Transverse to Fiber Normal to ply ($\sigma_{t}$ [S/m])
5500	203.5	20.9

**Table 2:** Distribution of the design variables along the domain.

$x_{i}$	Design Variable Description	Type	${x_{i}}^{L}$	${x_{i}}^{U}$
$x_{1}$	Crack Size, in (mm)	Continuous	5	30
$x_{2}$	Crack *x*-location, in (mm)	Continuous	4.5	83
$x_{3}$	Crack *y*-location, interlaminar	Discrete	1	15

**Table 3:** Effective Independence value groups for 2D models of $[0_{4}/90_{4}{]}_{s}$ and $[(0/90{)}_{4}{]}_{s}$ laminates and eight electrodes.

	Number of Groups and Group Description for Both [0_4_/90_4_]_s_ and [(0/90)_4_]_s_ Layups	
Ei Range	Number of Groups	Group description (Injection/Ground)
Ei ≥ 0.9	1	(2/6 , 3/7)
0.9 > Ei ≥ 0.5	2	(1/5 , 4/8) and (2/3 , 6/7)
0.5 > Ei ≥ 0.4	3	(2/7 , 3/6) ; (1/6 , 2/5 , 3/8 , 4/7) and (1/2 , 3/4 , 5/6 , 7/8)
0.4 > Ei ≥ 0.3	1	(1/7 , 2/8 , 1/3 , 2/4 , 3/5 , 4/6 , 5/7 , 6/8)
0.3 > Ei ≥ 0.2	1	(1/8 , 1/4 , 5/8 , 4/5)

**Table 4:** Summary of inverse identification results.

Layup	Number of Electrodes	Electrode Set	RMSE of Maximum Relative Errors (%)	Maxima of Maximum Relative Errors (%)	Number of Cases with RE(%)≥10%
${\left[0_{4}/90_{4}\right]}_{s}$	8	Full	10.42	50	6
	8	Reduced	8.09	37.5	6
	14	Full	5.11	25	5
	14	Reduced	6.02	27.38	5
${\left[{\left(0/90\right)}_{4}\right]}_{s}$	8	Full	5.22	19.43	5
	8	Reduced	7.65	33.78	6
	14	Full	5.31	32.69	1
	14	Reduced	3.37	16.09	1

RMSE: Root Mean Square Error; RE: Relative Error.

## Abbreviations

The following abbreviations are used in this manuscript:

CFRP	Carbon Fiber Reinforced Polymer
NDE	Non-Destructive Evaluation
ERT	Electrical Resistance Tomography
DOF	Degrees of Freedom
EI	Effective Independence
PCA	Principal Component Analysis
SVD	Singular Value Decomposition
BC	Boundary Condition
FEM	Finite Element Method
FEA	Finite Element Analysis
APDL	ANSYS Parametric Design Language
GA	Genetic Algorithm
DOE	Design of Experiments
RE	Relative Error
LHS	Latin Hypercube Sampling
RMS	Root Mean Square
RMSE	Root Mean Square Error
